# Authors’ Response to Peer Reviews of “Cross-Modal Sensory Boosting to Improve High-Frequency Hearing Loss: Device Development and Validation”

**DOI:** 10.2196/55510

**Published:** 2024-02-09

**Authors:** Izzy Kohler, Michael V Perrotta, Tiago Ferreira, David M Eagleman

**Affiliations:** 1 Neosensory, Los Altos, CA, United States; 2 Department of Psychiatry, Stanford University, Stanford, CA, United States

**Keywords:** audiology, hearing, high-frequency, wristband, develop, development, wearable, wearables, machine learning, phoneme, phonemes, hear, vibrotactile, vibration, vibrations, sound, sounds, hearing loss, loud noise, loud noises, noise pollution, hearing aids, hearing aid

*This is the authors’ response to peer-review reports for “Cross-Modal Sensory Boosting to Improve High-Frequency Hearing Loss: Device Development and Validation”.*

## Round 1 Review

We thank the reviewers for their very helpful feedback. Following their suggestions, we have clarified the language throughout and added several new figures and tables. Collectively, this has strengthened the manuscript and should address all concerns. Detailed responses below.

### Reviewer F [1]

*The authors report on an interesting study [2] in which they use a wearable device to sense high-frequency sounds. I have some specific comments below. To summarize, some essential elements are missing from the manuscript, and the manuscript needs significant editorial attention (errors, academic writing style, figures).*

*Introduction: I would suggest using primary references for the number of people with hearing loss (rather than Olusanya et al [3]) and for the burden of hearing loss (rather than Michels et al [4]). Regarding the risk of high-frequency hearing loss, have the authors overlooked the fact that this is commonly seen in most older adults (ie, what is attributed to aging)? This is mentioned in the second paragraph. The authors are mixing up noise-related hearing loss and age-related hearing loss (presbycusis) in the manuscript.*

Response: Thank you for all your comments. The Introduction was reworded for clarity. Additional references have also been added throughout the Introduction section. All is detailed below.

*Last sentence on the first page should not finish with a colon.*

Response: This has been replaced with a period.

*I do not think that Hickson et al [* 5 *] is a primary reference for limitations of hearing aids (HAs) and cochlear implants. “The auditory cortex is activated by vibrotactile information in individuals who are hearing impaired and deaf.” This implies that the auditory cortex is only activated this way.*

Response: This sentence has been revised to “The auditory cortex is primarily dedicated to the processing of sound, but can also be activated by vibrotactile information in individuals who are hearing impaired and deaf [6,7].”

*Middle paragraph: phonemes are extracted. How this is done should be provided here, not later in the manuscript.*

Response: All information regarding phoneme choice is presented together under the Algorithm section.

*Is the designation of the particular transducer important? In other words, is a larger temporal difference between the two most similar phonemes important?*

Response: This question was not tested in this research study; each phoneme was simply assigned to a different actuator. Our previous research [8] demonstrates that participants can learn to distinguish the spatial differences.

*“...unconsciously integrated...” Why is the word “unconsciously” needed?*

Response: With practice, the integration of vibrations with sound becomes automatic, not requiring constant awareness of which actuator is vibrating and the phoneme assigned to it.

*“The user is then able to understand...” Isn’t this yet to be shown, or is evidence provided in the next paragraph? If so, this needs to be made clearer.*

Response: We have added citations to previous published work to make this clear.

*Interestingly, the microphone is placed on the wrist, a part of the body that can often be situated away from the direct line of communication between two people (eg, under a table). Were the users trained to keep their wrists up?*

Response: Users were not trained to hold their wrist in a specific position; this was unnecessary because the microphone is omnidirectional.

*“. (Phatak et al., 2009) asked” and “s. (Sher & Owens, 1974) presented...”: references not properly incorporated into the sentences*

Response: These have been corrected, thank you.

*“...listening to an audiobook, podcast...” These are often streamed to personal headsets/earphones. Were any instructions provided in terms of volume, closeness to speakers, etc?*

Response: Participants were asked not to use headsets or earphones so that the microphone on the wristband would collect the sound. No further directions were given for volume or closeness to speakers. This has been clarified in the manuscript.

*Participants: Normally, information about participants is provided before most of the other information in a Methodology section, particularly before, for example, tasks.*

Response: The order of presentation within the Methods section has now been changed to place participants first.

*Abbreviated Profile of Hearing Aid Benefit (APHAB): I am not sure that I agree with the rationale that questions on aversiveness are not relevant. Cox and Alexander [9] write “Aversiveness of Sounds, quantifies negative reactions to environmental sounds,” and “The APHAB is a potentially valuable clinical instrument. It can be useful for quantifying the disability associated with a hearing loss and the reduction of disability that is achieved with a hearing aid.” That is, it is designed to be used before an intervention (and has been used a lot for non-HA interventions as well, eg, implants).*

Response: Thank you for the comment. We removed aversiveness questions from the APHAB because the haptic wristband does not alter or distort sound (as HAs can), and therefore, these questions did not directly apply to a wristband (eg, changing one’s tolerance for different types of sounds). We have clarified this in the manuscript.

*How was the APHAB administered?*

Response: The test was administered through an online questionnaire that captured the data onto a datasheet for analysis. This information has been added to the manuscript under “Abbreviated Profile of Hearing Aid Benefit (APHAB)”

*Please pay attention to tense when writing. In most cases, past tense should be used.*

Response: Done, thank you.

*How many male and female participants were in the study?*

Response: 10 males, 5 females, and 1 nonbinary. This information has been added to the Participant section.

*dB should be dB hearing loss.*

Response: This has been corrected.

*Using an audiogram from any mobile-based device means little guarantee of accuracy.*

Response: We have now clarified in the manuscript that smartphone hearing apps (eg, Mimi; which we used) has been found to be comparable to in-clinic testing (eg [10]).

*What was the rationale for the specifications for the audiogram?*

Response: This was simply a general inclusion criterion to make certain we were capturing garden-variety presbycusis.

*“ear’s” should be “ears.”*

Response: corrected

*Any reason why 16 people were recruited?*

Response: As a general rule, we consider 10 subjects a minimum number for a good psychometric study. In this case, we recruited 19, and 3 dropped out. Our retrospective power analysis shows that 16 participants were well sufficient given the outcome magnitude.

*What is “(11.6),” SD? And “(13)” and “(9)”?*

Response: Yes, we have now clarified this in the manuscript.

*Figure 3: I suggest not including the values on the plot. Furthermore, “Error boundary represents standard error of the mean.” The reader has to interpret the “error boundary” as the gray area.*

Response: We would prefer to keep the values in the plot, as more information is better. However, we have clarified the definition of error boundary in the figure caption.

*“...to drop at a slower, more steady pace for the remaining five weeks of the study.” Writing could be tightened up a bit, and there is a rise in scores at 3 weeks. If the response is that there is not a significant increase, then it would be good to report at what point the difference is not significant.*

Response: This was reworded to “The average aided APHAB score continued to trend down for the remaining 5 weeks of the study.” The wording was chosen so as not to imply that continued improvement stopped after a week.

*Regression analysis: This is OK, but the use of a paired sample t test could have been taken for both analyses.*

Response: There may be a misunderstanding here. The regression analysis in Figure 6 of the final paper simply characterizes the relationship between baseline score and final outcome. A *t* test would not be possible here.

*On the other hand, a multinomial regression analysis could have considered the influence of age, HA user or not, or baseline APHAB scores on final APHAB scores.*

Response: Thank you for the suggestion. Unfortunately, our sample size is not sufficiently large enough to yield good signals from a multinomial regression, especially as some of the suggested categories (“HA user or not”) are binary. We will keep this in mind for future studies as our sample size grows.

*I see that there were approximately equal numbers of HA and non-HA users. Was this by accident or design? It is not mentioned in the Recruitment section.*

Response: The approximately even numbers was hoped for but fortuitous.

*Do not start sentences with “Also.”*

Response: We have replaced with “additionally.”

*Figure 6: It is not clear which score is being reported. At 6 weeks? I suspect it means the difference between the baseline and final scores. If so, this needs to be made clear in the caption.*

Response: The caption has been corrected for clarification; the graph represented 6 weeks.

*It appears that there was no attempt to record the listening environments of the users nor how often they used their devices.*

Response: We have added the following segment to the Results section:

“Time wearing the wristband and time exposed to speech was verified through collection of data from backend logging that records when the wristband is turned on or off and when a phoneme is detected. As seen in Figure 5 participants wore the wristband for and average of 12.9 (SD=8.1) hours per day and were exposed to speech for an average of 6.7 (SD=3.3) hours per day.”

*“One potential hypothesis” should be “One potential explanation.”*

Response: This has been corrected.

*“Participants without hearing aids benefitted the most from vibrotactile sensory substitution...” True—in fact, those with HAs did not get significant benefits.*

Response: Thank you. This is now described in detail in the Results section.

*It is always good to devote a bit of space to the limitations of the study. This is missing in this manuscript.*

Response: Thank you. A limitations paragraph has now been added.

*“Future studies will focus on quantifying the maximum benefits possible and how long improvements continue before a plateau is reached.” This is not a conclusion of the study.*

Response: Thank you. This line was removed from the Conclusion.

*Perhaps this is mentioned elsewhere, but the device is given a name; it would be good to know about the association between the authors and the manufacturer of the device.*

Response: The authors are associated both with Stanford University and the company Neosensory, which makes this device. This information is in the paper.

### Anonymous [11]

#### General Comments

*This paper highlights the utility and perceived communication benefits of the Clarity vibrotactile band for users with high-frequency hearing loss. Overall, this is a well-designed study that demonstrates the effectiveness of this assistive listening device that provides benefits for listeners with high-frequency hearing loss in complex listening situations as measured by the APHAB. Additionally, this study provides subjective evidence that both HA users and non-HA users experience benefit from the Clarity device. Specifically, the non-HA users report more benefits across different listening conditions (background noise [BN] and reverberation) than HA users.*

#### Major Comments

1. *Consider referencing Glick and Sharma [12] in your Introduction as it relates to the cross-modal plasticity associated with age-related hearing loss (presbycusis).*

Response: This reference has been cited. Thank you for making that recommendation.

2. *In the Methods section, consider starting with a clear description of the participants. Who are they, how many, how many were HA users versus non-HA users, age, etc. While the majority of this information is embedded later in the article, it is not readily accessible.*

Response: A demographic chart was added to the manuscript that outlines the important demographic characteristics of all of the participants.

3. *In the Methods section, consider creating a subheading or table for the audiometric data of the participants and including additional information like a description of their audiometric data (type, degree, configuration), pure tone average (500, 1000, and 2000 Hz), symmetry of the hearing loss, how many were considered to be within normal limits up to 2000 Hz versus having hearing loss at lower frequencies (≤2000 Hz). This could have a significant impact on speech understanding difficulties, especially in complex listening environments.*

Response: A chart has been added to the supplementary materials (see Table 1 in final paper); it includes all audiometric data for the participants.

4. *For the audiometric data, how many participants provided their test results from a doctor of audiology or hearing health care professional? How many provided results from the mobile app? Is it possible to confirm that all participants had sensorineural hearing loss and not mixed or conductive hearing loss?*

Response: A total of 7 participants provided 2 audiograms from the online assessments (a Mimi hearing assessment), and 9 provided audiograms from an audiologist. The type of hearing loss was not confirmed; this has been added to the Limitation section of our Conclusion.

5. *In the Device subsection, consider adding additional information regarding the microphone characteristics. Additionally, define “GRMS.”*

Response: A table was added to the supplementary materials (Multimedia Appendix 1 in final paper).

6. *In the Algorithm subsection, you mention the sham algorithm and the /f/ motor. In the sham condition, which motor represents the /f/ phoneme, and which additional phonemes are used in the sham condition?*

7. *Additionally, the sham condition is never mentioned in the Results or Discussion. Consider adding this information to the manuscript, or if you choose not to, consider not introducing the sham algorithm.*

Response: Oops, that sentence was mistakenly included from a previous internal study. We have fixed this now, removing the description of the sham algorithm. For clarity, in this experiment, a sham was not used.

8. *In Figure 3, consider changing the y-axis to “APHAB Score (%)” and refer to the APHAB benefit scores as scores or percentages instead of points in the text.*

Response: The standard method of interpreting the APHAB is to look at unaided (baseline), aided (final), and benefit scores (unaided – aided). Please see the following paper for more details:

Cox RM. Administration and application of the APHAB. *Hearing J.* Apr 1997;50(4): 32. [doi: 10.1097/00025572-199704000-00002]

9. *For the simple linear regression, consider adding a statement that indicates what this means or its importance.*

Response: This was reworded for clarification: “Simple linear regression analysis was used to test if a participant’s baseline APHAB score explains their benefit APHAB score after 6 weeks, indicating that those with greater subjective difficulty understanding speech may stand to benefit the most from the haptic assistance of the wristband.”

10. *In Figure 5, consider adding bars for weeks 0 and 1 to help readers visualize the results in the text.*

Response: Thank you for this suggestion; we have added this as Figure 7 in the final paper.

11. *Consider creating a line graph that highlights the greater decrease in APHAB scores from baseline to week 6 for those without HAs than those with HAs (as discussed in the Results).*

Response: Thank you for this suggestion. The graph we added (Figure 7 in the final paper) highlights the difference as per your request.

12. *In Figure 6, this figure represents benefit scores from baseline (wk 0) to week 6, correct? Consider clarifying the figure text and removing the information regarding the subgroups.*

Response: We further clarified the figure in the caption. We prefer to keep the subgroups represented in the caption to illustrate what is further described in the text.

13. *In the Discussion and Conclusion sections, I do not think it is accurate to say that the Clarity device “improved their understanding of speech communication” because that was not what was measured. The APHAB is a subjective measure, which to me means that all the benefits users received from using the Clarity are perceived benefits and are not measurable improvements in understanding. To claim speech understanding improvements, I feel you would need to document that through an objective speech understanding measure such as the word recognition score in quiet, word recognition score in noise, Quick Speech in Noise, etc.*

Response: The Discussion and Conclusion were reworded to clarify the subjectivity of the APHAB and what the results indicate. For example, in the Discussion, we have rephrased our sentence to say “Here, we demonstrated that individuals with high frequency hearing loss are able to improve their subjective understanding of speech communication using vibrational representations of high frequency speech sounds on the wrist.”

14. *In the Discussion section, you refer to the group with a higher APHAB score experiencing a greater improvement. Is this the group that uses HAs, or is this a different subgroup? It would be interesting to know how many in this group had hearing loss between 250-2000 Hz.*

Response: This was referring to the subgroup that started the study with a higher baseline score. This clarification has been added to the sentence.

15. *In the Discussion section, you report subgroup data for BN, reverberation, and ease of communication (EOC) that is not documented or reported in the Results section or any figures/tables. Consider adding this.*

Response: The scores referred to in the Discussion are all reported in the Results section. Figure 8 in the final paper is the accompanying graph.

16. *In the Conclusion section, you mention that “results also demonstrate that individuals who had the greatest amount of difficulty understanding speech prior to.” Is this the without HA subgroup or a different subgroup? A few times throughout the article, these labels appear to be used interchangeably. While this may be accurate for your data set, I would caution that these terms/labels are not mutually exclusive.*

Response: Those who had the greatest amount of difficulty understanding speech prior to starting the trial refers to those who started the study with the highest APHAB baseline score. The line in question has been reworded to:

“Finally, our results also demonstrated that those who started the study with a higher APHAB score (greater hearing disability) experienced the greatest amount of benefit from vibrotactile feedback.”

#### Minor Comments

1. *In the Introduction, the authors mention that HA and cochlear implant users commonly report disappointment with understanding speech and reference Hickson et al [5]. While this could be true, the majority of users’ complaints are specifically related to difficulties understanding speech in complex or noisy listening environments, not just in quiet as is implied.*

Response: This sentence has been changed to “One of the most commonly reported disappointments among users of HAs and CIs is that they still cannot understand speech, especially in complex environments.”

2. *How much were participants compensated for their participation?*

Response: Participants were given a US $100 gift card for their participation. This is now clarified in the manuscript.

3. *In Figure 2, I assume your scale for the y-axis is dB of hearing loss? Consider clarifying which dB scale was used.*

Response: Thank you, this has been corrected.

4. *In the Paradigms subsection, does the Clarity device have any data logging features that can objectively record how often or how long the participant is using the device or in what listening conditions the user is in with the device (eg, quiet rooms, noisy restaurants, or reverberant auditoriums)?*

Response: A usage graph (Figure 5 in the final paper) has been added.

5. *In the APHAB subsection, consider rewording for clarity: “modified version of the Abbreviated Profile of Hearing Aid Benefit (APHAB) which did not include six questions related to the aversiveness subscale (Cox, 1997).”*

Response: We have changed the wording, thank you.

6. *In the Results section, consider rewording for clarity: “...they ended the study at a lower level of disability than those with hearing aids.”*

Response: We have reworded the sentence, thank you.

7. *The implication of microphone location briefly mentioned in the Discussion is very important in my opinion. Microphone location is a significant issue even for ear-level HAs. I can only imagine the microphone placement significantly impacts the benefit and utility of the Clarity.*

Response: This was added as a limitation of the study.

8. *In the Conclusion section, consider rewording for clarity: “We found that while both hearing aid and non-hearing aid users with high frequency hearing loss reported benefited, vibrotactile feedback appears to be more beneficial for non-hearing aid users.”*

Response: Done, thank you for the suggestion.

9. *The manuscript does not include an ethical approval statement or a limitations section.*

Response: Ethical Approval section has been added.

“The study protocol was approved by Solutions IRB, an independent institutional review board accredited by the Association for the Accreditation of Human Research Protection Programs, Inc. All subjects gave written informed consent in accordance with the Declaration of Helsinki.”

Limitations section has been added:

“There are limitations of this study. First, the small sample size prevents extrapolation of the results to larger populations; this will be addressed in future studies. We were also limited in our ability to collect speech comprehension data in a noise-controlled environment with standardized volume controls – this is because the testing was done in participant homes instead of a laboratory. As a result, this study depended on self-report data (APHAB) which always has the potential of being influenced by a placebo effect. Another limitation is that some participant audiograms were assessed via phone applications rather than an audiologist’s office; however, it should be noted that these appear to yield roughly equivalent results [13]. We also note that the specific type of hearing loss was also not controlled for beyond meeting the audiogram requirements. One final thing to note is that participants could move their hand (and hence their wristband), meaning that the microphone placement was not standardized in a single position. We do not consider this a limitation of the study, as the study is meant to test whether a vibrotactile wristband can be used to detect sound. The positive results reported here suggest that the mobility of the microphone does not present a problem.”

### Anonymous [14]

#### Overall

*This study reports on an interesting device with intriguing clinical implications for people with hearing loss.*

*Innovative, and worthy of reporting on this technology, which could inspire other researchers*

*But there are some issues that I feel require revisions:*

- *Conflation of self-reported and objective benefit in the write-up*
- *Lack of reporting the range and dispersion of the data—paper focuses on group means and gives very little ability to draw any inferences about individual participant variability*
- *Lack of objective data about performance of the algorithm and participant performance for speech understanding*

Response: Objective data for algorithm performance has been added.

- *No data presented for the final questionnaire presented in the Methods*
- *Presentation and discussion of results switches back and forth between benefit scores and raw scores in a way that is unclear and makes the paper difficult to follow and interpret at times*
- *Some conclusions are presented without statistical results to support them*
- *Some conclusions are stated too strongly given the sample size and study design*
- *Lack of a limitations section to help reader contextualize the results*

#### Abstract

- *“...improve their understanding of verbal communication.”: Please indicate that this is a self-reported or self-perceived understanding of verbal communication. I think it is important to distinguish the results from objective speech recognition testing (acknowledging that self-reported benefit is very important).*

Response: We have modified the wording to emphasize the subjective nature of the APHAB.

- *“...greatest amount of benefit...”: Please indicate that it is a self-reported or self-perceived benefit.*

Response: We have modified the wording to emphasize the subjective nature of the APHAB.

#### Introduction

*Page 2: Authors indicate that auditory and vibrotactile information can be unconsciously and naturally integrated in the brain. It would be helpful if the authors could give some description/details of how the integration is hypothesized to occur—how long it takes and what neural/cognitive mechanisms might support it. Even if this is just a hypothesis, it would provide helpful context.*

*Page 3: “...can help individuals with high frequency hearing loss better understand speech communication...” Please indicate that it can help them self-report or self-perceive better speech communication.*

Response: Reworded to “In this study, we demonstrate that a simple wearable sensory substitution device that transforms speech sounds into haptic vibrations on the wrist can help individuals with high frequency hearing loss to feel more confident in their ability to understand speech communication throughout their normal daily routine.”

*Page 3: The last sentence is too strong. A device can help improve self-reported speech communication without translating to the types of benefits the authors describe in these various situations/environments. It could say something like “the evidence demonstrate the promise of this technology, which if further developed and refined holds promise for...”—something like this. I think it is OK to indicate that these kinds of benefits are possible in the future, but they are not directly supported by the results of this small study. Lots more work is needed.*

Response: Reworded to “With further development and refinement, this technology has the potential to improve the quality and productivity of their daily interactions, enable them to enjoy audio based entertainment such as movies and podcasts, help them understand conversations in complicated acoustic environments, and fill the residual gaps of impairment left by their hearing aids.”

#### Methods

*Not much detail about the machine learning algorithm is provided. More detail about how it filters BN and identifies phonemes would be helpful. How was the algorithm trained? Assuming it was trained on speech, what regional accents were used?*

Response: We have added the following details to the Methods section:

“The phoneme detection algorithm was trained using the elastic compute cloud on Amazon Web Services (AWS). The training data consisted of a combination of pure LibriSpeech and Librispeech re-recorded through the onboard microphone on the wristband. Librispeech is a corpus of approximately 1000 hours of English speech with standard American accents sampled at 16 kHz that has been shown to produce excellent performance in speech recognition models trained with it [15]. To produce a corpus of English read speech suitable for training speech recognition systems, Librispeech aligns and segments audiobook read speech with the corresponding book text automatically and then filters out portions with noisy transcripts. The purpose of using re-recorded data was to tune the algorithm’s parameters to speech sounds representative of those it would encounter from the wristband’s microphone.”

*Related to the above, it is not clear how the sham algorithm was used in developing the algorithm. Additional detail/description would be helpful.*

Response: There was no sham algorithm in this study. That sentence was inserted mistakenly and has been removed.

*Page 4: The authors mention that the algorithm performed poorly for some consonants that people with hearing loss have trouble hearing. It is not clear what level of performance constitutes poor performance and what level constitutes good performance for the phonemes that were selected for the algorithm. More context here would help the reader to understand the results. Understanding the algorithm’s accuracy is important for contextualizing the users’ results. It would be reasonable to suspect that the users’ results should be closely linked to the algorithm’s accuracy.*

Response: The specifics of the machine learning algorithm’s performance have been included in Table 2 in the final paper.

#### Tasks

*I am a bit confused as to why objective speech recognition testing was not completed. The self-reported benefit is absolutely important, but based on the Introduction, the reader is interested in knowing how objective speech recognition improved with the wristband for the selected consonants. If these data are available, it would be helpful to add them. If not, it would be helpful if the authors could explain—somewhere in the manuscript—why this testing was not completed/reported.*

Response: This information was added to our Limitations section: “We were also limited in our ability to collect speech comprehension data in a noise-controlled environment with standardized volume controls. This is because the testing was done in participant homes instead of a laboratory. As a result, this study depended on self-report data (APHAB), which always has the potential of being influenced by a placebo effect.”

*Final questionnaire: It does not seem like the results of the final questionnaire are reported in this manuscript. Given that the APHAB is the only reported outcome measure, it would be helpful to add these results as well, as they represent something more holistic than the weekly APHAB results.*

Response: Three of our participants requested to continue use of the wristband after the study ended, and hence, they did not fill out the final questionnaire. Of those who did, some had criticisms (“I’m really unsure if the Clarify band was helpful or not”) and some had praise (“It was very beneficial. Thank you”); however, the comments were too few to be statistically meaningful. This information has been added to the Results section.

#### Paradigm

*Does the wristband provide any data logging to indicate how many hours per day the devices were worn? If not, this is not a major flaw but should be mentioned as a limitation because it seems like wear time could directly affect benefit.*

Response: We have added the following segment to the Results section:

“Time wearing the wristband and time exposed to speech was verified through collection of data from backend logging that records when the wristband is turned on or off and when a phoneme is detected. As seen in Figure 5, participants wore the wristband for and average of 12.9 (SD=8.1) hours per day and were exposed to speech for an average of 6.7 (SD=3.3) hours per day.”

#### APHAB

*It might be helpful to the reader to clarify that higher raw APHAB scores indicate worse performance and lower scores indicate better performance but higher benefit scores represent more benefit or better outcomes.*

Response: We added this line to the APHAB section: “Lower raw APHAB scores indicate lower levels of disability associated with hearing loss. Higher benefit scores indicate more perceived benefits from intervention.”

#### Participants

*I would suggest adding the number who did and did not use HAs in this section. Any additional information regarding participants—gender, education, etc—would be helpful if it is available to report. Otherwise, I suggest adding that a limitation of the paper is the limited demographic information of the participants (combined with a small n), which makes it hard to determine if any participant-level characteristics might influence the benefit of the wristband.*

Response: We have added a demographic table to the paper indicating demographic information for each participant: age, gender, HA use, years with hearing loss, and hearing loss profile (Table 1 in the final paper).

*The authors mention that if a clinical audiogram was unavailable, participants completed an audiogram via a mobile app. Then the authors provide an example, Mimi. Did everyone who used a mobile app use the Mimi app, or did some use other apps?*

Response: The two tests used were Mimi [16] and the Hearing Test & Ear Age Test [17]. Participants who did not have an audiogram from an audiologist were required to provide audiograms from both apps. This was clarified under the Participants section.

*Relatedly, it would be helpful to report how many participants had clinical audiograms and how many used an app to provide context for the audiometric results.*

Response: This line was added under the Participants section: “Nine participants provided audiograms from an audiologist and seven provided audiograms from the 2 mobile apps.”

#### Results

*One critique is that I did not feel like I got a very good handle on the descriptive statistics before the authors started showing group means (with SE of the mean) and comparisons (both over time and between subgroups of participants). I felt that the results emphasized group means (with SE of the mean), but I did not get a good sense of the range and dispersion of the data. In the Discussion, the authors start discussing the numbers of participants who started or ended at a specific APHAB overall score range, but I did not feel like I had the information in the paper to help me contextualize that discussion (because the results, as presented, do not give a very clear view of how individual participants may have performed).*

Response: Thank you for this. We have now added tables in the supplementary material (Multimedia Appendices 2 and 3 in the final paper).

*To address the above point, I would strongly suggest adding a descriptive results table first that gives the means, maximums and minimums, and SDs for the overall APHAB scores and, possibly, APHAB benefit scores. It would be nice to see these values for the full participant group, as well as for the subgroups of participants with and without HAs (including the n in each group). It would be helpful to see the same data for the subscale scores (EOC, BN, reverberation) if it fits in the table, but I think the overall APHAB scores would be sufficient if space is an issue. Another consideration is that with only 16 participants, you could show the individual-level data for each participant, who would each be a row, and then give the group data in a different row. I defer to the authors on their preferences but would simply suggest that some revisions be made to give the reader a better grasp of the descriptive results.*

Response: Done—see above.

*In the section where subscale analyses are given, the write-up describes comparisons of subscale benefit scores between the different subgroups (with and without HAs) as well as comparisons, within a subgroup, of benefit scores to the baseline score. Throughout this section, it is hard to track which P values go with which comparisons. It is hard to read and interpret. Additionally, the information is presented slightly differently for each subscale, which makes it even harder to follow. Clearer written descriptions of each comparison being tested—then followed by the statistical numbers—would be beneficial for the reader. Additionally, using a parallel results presentation for each subscale would be helpful.*

Response: Thank you for the suggestions. This section was reworded with better consistency and clarity.

“Subscale analyses were performed for ease of communication (EOC), background noise (BN), and reverberation (RV) (Figure 8 and Supplemental Table 2). These subscales are reflective of speech communication under ideal conditions, in noisy environments, and in reverberant environments. The average benefit score for EOC was 15.44 (SD=13.88 , *n*=16, *P*<.001 , two-tailed dependent t-test). Those who wore hearing aids and those who did not wear hearing aids had similar EOC benefit scores (t(14)=2.18, *P*=.6, two-tailed independent t-test). The average EOC benefit for those with hearing aids was 13.57 (SD=15.71, *n*=9, *P*=.03, two-tailed dependent t-test) and the average EOC benefit for those without hearing aids was 17.83 (SD=11.85, *n*=7, *P*=.01, two-tailed dependent t-test). The average benefit score for BN was 10.88 (SD=17.54, *n*=16, *P*=.03, two-tailed dependent t-test). The average BN benefit for those without hearing aids was 16.99 points higher than those hearing aids (t(14)=2.14, *P*=.05, two-tailed independent t-test). The average BN benefit for those with HA was 3.44 (SD=17.5 , *n*=9, *P*=.54, two-tailed dependent t-test) and the average BN benefit for those without hearing aids was 20.43 (SD=15.1 , *n*=7, *P*=.01, two-tailed dependent t-test). The average benefit score for RV was 10.84 (SD=16.95, *n*=16, *P*=.02, two-tailed dependent t-test). The average RV benefit score for those without hearing aids was 11.12 points higher than those with hearing aids (t(14)=2.14, *P*=.20, two-tailed independent t-test). The average RV benefit for those without hearing aids was 17.10 (SD=16.0 , *n*=7, *P*=.03, two-tailed dependent t-test) and the average RV benefit for those with hearing aids was 5.98 (SD=17.0 , n-9, *P*=.32, two-tailed dependent t-test).”

#### Discussion

*“...individuals with high frequency hearing loss are able to improve their understanding of speech communication...”: I would like to see it be specified that this is an improvement in self-reported or self-perceived understanding of speech communication. Previous HA research shows there can be a placebo effect associated with the perception that one is wearing advanced technology [18].*

Response: Language has been changed throughout the document

*“...participants were able to improve their ability to understand conversations during daily interactions.”: Same comment as above. Please indicate this is a self-reported or self-perceived ability to understand conversations.*

Response: Language has been changed throughout the document to “Here, we demonstrated that individuals with high frequency hearing loss are able to improve their subjective understanding of speech communication using vibrational representations of high frequency speech sounds on the wrist.”

*“We further found that participants who started the study with a higher APHAB score experienced a greater improvement in their ability to understand speech by the end of the six week trial.”: As mentioned earlier in this review, this result is hard to interpret without any sense for the individual variability in the data. The results are presented as group means without clear maximums and minimums or SDs. Providing this information in the Results would help give context to this claim in the Discussion.*

Response: As above, we added two tables with this information to the supplementary material (Multimedia Appendices 2 and 3 in the final paper).

*“Out of 16 participants, 14 ended the study with an APHAB score of 40 or below...”: This is again difficult to interpret without any sense for the individual-level data. At timepoint zero, the group mean is right around 40. It is not clear if ending the study at 40 or below indicates benefit or is just a reflection of peoples’ starting points. The discussion should be framed in terms of the amount of benefit people reported.*

Response: A graph (Figure 4 in the final paper) was added to the Results section.

*“Five participants started the study with an unaided APHAB of 50 points or higher...”: Again, a better sense of the individual-level data and dispersion would help give context for this. The Results are focused on means and then the Discussion brings up individual data, and it is hard to interpret the two together.*

Response: See the supplementary tables (Multimedia Appendices 2 and 3 in the final paper).

*A small point but should this be <30 not >30 as written in the text?*

Response: No, the >30 benefit score is correct.

*“One potential hypothesis...”: It seems like this could also be due to having more room to improve their everyday speech understanding. I think it is important to acknowledge possible noncortical factors that could explain this finding (though I think it is fine to also leave the possibility that it reflects cortical characteristics).*

Response: This was reworded to “One potential explanation for why participants who started the trial with greater difficulty understanding speech experience greater improvement is that more of their auditory cortex is available for the interpretation of tactile sound representation (Auer et al ., 2007). It is also possible that participants who started the study with a lower APHAB score had more room for improvement. This could be an interesting topic for future research.”

*“Participants without hearing aids benefitted the most...”: I think given the lack of statistical significance in the comparison of the group means, this needs to be toned down a bit. Perhaps something like “Participants without hearing aids demonstrated a trend toward higher self-reported benefit, though this did not reach statistical significance.” I know the authors reference the Cox 10-point criterion, but I am not sure that can be accurately applied to these data when the statistical test says the group means themselves are not statistically different (maybe related to the small sample size and variance in the data). Again, I would also like the benefit to be specified as self-reported or self-perceived.*

Response: This has been changed to “Participants without hearing aids demonstrated a trend toward higher self-reported benefit from vibrotactile sensory substitution for speech understanding, though this did not reach statistical significance.”

*“Given that this group started the study with a higher APHAB score...”: I did not find where there is a statistical test to justify this claim. This should be justified with a t test. Otherwise, I think it would be OK to specify that the t test did not show a statistical difference, but this group is trending toward having a higher baseline APHAB score.*

Response: This was reworded to “Given that this group started the study trending toward a higher APHAB score (above), we presume the difference is because the hearing aid group already gains benefit from their technology and therefore has less room for improvement.”

*“In this study, we demonstrated the addition of vibrotactile feedback in the presence of background noise enabled individuals who did not wear hearing aids to hear speech communication better...”: Again, would like to see it noted that this is a self-reported or self-perceived benefit.*

Response: This was reworded to “In this study, we demonstrated the addition of vibrotactile feedback in the presence of background noise enabled individuals who did not wear hearing aids to hear speech communication better based on their subjective experience.”

*The authors present the final average BN scores (eg, 28.95 and 40.04), but the section above seems to be focused on benefit scores. This reflects my earlier comment about providing more descriptive data upfront. It is hard to track how the authors switch between baseline and benefit scores, and without a descriptive table to refer to, it is difficult to contextualize some of the Discussion.*

Response: The benefit score is the baseline score minus the final score. The supplementary table (Multimedia Appendix 3 in the final paper) should help to clarify this.

*Related to the above, these scores are presented as being different but are they statistically different?*

Response: Indeed, they were statistically significant. This is clarified in the Results section:

“The average benefit score above baseline for BN was 10.88 (SD=17.54, *n*=16, *P*=.03, two-tailed dependent t-test), with a 16.99 point difference in BN benefit between those who wore and did not wear hearing aids (no hearing aids 20.43 benefit, hearing aids 3.44 benefit, t(14)=2.14, *P*=.05, two-tailed independent t-test).”

*“...suggesting that those who use hearing aids may benefit from using vibrotactile feedback during conversations in background noise instead of using their hearing aids.”: I think this is much too strong of a conclusion for the data, study design, and sample size. This needs to be significantly toned down—as written, I think this is a reckless conclusion based on the limitations of the data. I could be OK with presenting this as an interesting finding worth future research to determine if the above could potentially be true. However, it would need to be framed by saying that this sort of clinical recommendation would require much larger, more rigorous studies with blinding of participants and researchers.*

Response: This line was added for clarification: “While our data do not offer conclusive evidence of this due to several limitations, it does offer an area worth further exploration in larger studies.”

*“Similar to our findings in background noise, we also found...”: From what I can see in the subscale results discussion, the difference between the group with and without HAs did not reach statistical significance. If this is true, it seems to be going too far to say that the wristband helped people without HAs the most. Here, I think it is OK to note that the results are trending in this direction as long as it is acknowledged that the results did not reach statistical significance.*

Response: This has been rephrased: “Here we found that the addition of vibrotactile haptic vibration to the wrist in reverberant environments tended to help the participants without hearing aids more than those with hearing aids, though the difference did not reach statistical significance.”

*“At the end of the trial, the group of participants who did not wear hearing aids showed an average reverberation score that was less than the average for the group who were regular hearing aid users.”: Was this tested statistically? From what I can tell, it looks like only the benefit scores are presented in the Results—not the raw scores. If the Discussion brings up the raw score (not the benefit), this should be presented in the Results section. Again, statistical results are needed to draw conclusions regarding the comparison of means.*

Response: This sentence was consolidated with the prior sentence (above).

*“It is possible that individuals who use hearing aids may find haptic vibrations to be more helpful in reverberant environments...”: Similar to a comment above, I could be OK with presenting this as an area for future research, but I think it needs to be framed by noting the limitations of this study for drawing any clinical recommendations around HAs versus haptic vibration.*

Response: This was reworded: “One possibility to be tested is that individuals who use hearing aids may find haptic vibrations to be more helpful in reverberant environments when the hearing aids are removed because it would eliminate any conflict between the digital processing of the hearing aid and the vibrational signals that are providing information about the sounds of speech without processing.”

*“Upon completion of the trial, the average EOC score...”: Similar to previous comments, it seems that the Results only present benefit scores but now the Discussion mentions raw EOC scores for the group with and without HAs. If raw scores are mentioned in the Discussion, they should be presented in the Results.*

Response: The tables added to the supplementary material (Multimedia Appendices 2 and 3 in the final paper) will now clarify this.

*This section ends by noting equivalent ending EOC scores for the group with and without HAs; a statistical result should be presented to make this claim (and should be presented in the Results section).*

Response: Independent *t* test results are located in the Results section.

*One additional note: Results from the final questionnaire do not seem to be presented. Is there a reason for this? Given that the APHAB is the only outcome measure, it would be beneficial to see results from the final questionnaire in this paper alongside the APHAB. The final questionnaire also measures something a little different than the APHAB—it is more holistic for the whole field trial experience.*

Response: Three of our participants requested to continue use of the wristband after the study ended, and hence, they did not fill out the final questionnaire. Of those who did, some had criticisms (“I’m really unsure if the Clarify band was helpful or not”) and some had praise (“It was very beneficial. Thank you”); however, the comments were too few to be statistically meaningful. This information has been added to the Results section.

#### Conclusion

*Same comments as before about noting that this study applies to self-perceived or self-reported benefit.*

*“We found that vibrotactile feedback provides more benefit for those without hearing aids than for those with hearing aids...”: From what I see in the Results section, the statistical results do not support this conclusion. The 10-point criterion from Cox cannot be applied if we are not sure the group means themselves are even different (as indicated by the insignificant P value). I think it is OK to say the data are trending in this direction and that the small n may render the study underpowered to detect this difference at P<.05. Future work is needed to establish whether this claim is true. For now, I would argue it needs to be softened based on the findings and limitations of the study design.*

Response: This sentence was changed to “We found that vibrotactile feedback tends to provide more benefit for those without hearing aids than for those with hearing aids, although it does provide benefit for both. The small sample size may have rendered the study underpowered to detect this difference at *P*<.05 and further study is necessary to validate this finding.”

*Finally, I suggest adding a limitations section, which could note limitations around:*

- *Small n*
- *Reliance on self-report data without objective speech-testing data*
- *Potential for placebo effect to influence results*
- *Small n makes it difficult to discern whether/how individual and demographic characteristics could affect ability to integrate the haptic vibrations and benefit from the wristband—some characteristics one might wonder about include baseline cognitive ability, education level, differences in underlying degree/configuration of hearing loss, or duration of hearing loss*
- *Use of nonclinical audiogram for some participants (a minor limitation but should be noted)*
- *No information on how many hours per day the wristband was worn. One might hypothesize that outcomes could be related to wear time. Furthermore—beyond raw wear time—we also do not have information about the richness/complexity of auditory information processed through the wristband*

Response: The following Limitations section was added:

“There are limitations of this study. First, the small sample size prevents extrapolation of the results to larger populations; this will be addressed in future studies. We were also limited in our ability to collect speech comprehension data in a noise-controlled environment with standardized volume controls – this is because the testing was done in participant homes instead of a laboratory. As a result, this study depended on self-report data (APHAB) which always has the potential of being influenced by a placebo effect. Another limitation is that some participant audiograms were assessed via phone applications rather than an audiologist’s office; however, it should be noted that these appear to yield roughly equivalent results [10]. We also note that the specific type of hearing loss was also not controlled for beyond meeting the audiogram requirements. One final thing to note is that participants could move their hand (and hence their wristband), meaning that the microphone placement was not standardized in a single position. We do not consider this a limitation of the study, as the study is meant to test whether a vibrotactile wristband can be used to detect sound. The positive results reported here suggest that the mobility of the microphone does not present a problem.”

## Round 2 Review

### Reviewer F

#### General Comments

*The authors appear to have responded to previous comments. However, having two different versions of the manuscript in the system has caused confusion. Having some sort of system to track changes would also have been very useful.*

*The authors have persisted with using Michels et al [* 4 *]; this is not a primary reference for results of noise exposure of burden of hearing loss.*

Response: We have added two more additional references to support the claims of noise exposure causing hearing loss in the higher frequency ranges. Both of these references have been cited in over 100 publications:

- Chen KH, Su SB, Chen KT. An overview of occupational noise-induced hearing loss among workers: epidemiology, pathogenesis, and preventive measures. *Environ Health Prev Med*. Oct 31, 2020;25(1):65. [doi: 10.1186/s12199-020-00906-0] [Medline: 33129267]
- Hong O, Kerr MJ, Poling GL, Dhar S. Understanding and preventing noise-induced hearing loss. *Dis Mon*. Apr 2013;59(4):110-118. [doi: 10.1016/j.disamonth.2013.01.002] [Medline: 23507351]

*I am not convinced that even an omnidirectional microphone would be optimally placed on the wrist.*

Response: Thank you for your recommendation. The wrist placement was a decision made based on practicality for the user. In the past, we tried various form factors (including a vest), but those turned out to be impractical for daily use. During the algorithm design, different listening conditions were accounted for in the training data. In the end, our data make it clear that the current form factor works well; the future will tell if there is another form more optimal.

*“...allow them to enjoy audio based entertainment such as movies and podcasts...” was of course not tested.*

Response: We were not making a declaration in this sentence—we were simply identifying potential implications of improving one’s ability to understand speech. (“With further development and refinement, this technology has the potential to improve the quality and productivity of their daily interactions, enable them to enjoy audio based entertainment such as movies and podcasts, help them understand conversations in complicated acoustic environments, and fill the residual gaps of impairment left by their hearing aids.”)

*The last paragraph of the Introduction reads like a conclusion, not the presentation of aims or objectives.*

Response: We have revised the last paragraph of the introduction to now read “In this study, we aimed to demonstrate that a simple wearable sensory substitution device that transforms speech sounds into haptic vibrations on the wrist can help individuals with high frequency hearing loss to feel more confident in their ability to understand speech communication throughout their normal daily routine.”

*I am unconvinced about the rationale for removing aversiveness from the APHAB; the same can be said about the other subscales. It is not about the unpleasantness introduced by the device; otherwise, why should the APHAB be applied before an intervention such as HAs or cochlear implants (as done in this study)? It is the person’s overall aversiveness to sound. Anyway, the data were not collected, so there is little to be done.*

Response: The following questions address aversiveness in the APHAB:

- Unexpected sounds, like a smoke detector or alarm bell are uncomfortable.
- Traffic noises are too loud.
- The sounds of running water, such as a toilet or shower, are uncomfortably loud.
- The sounds of construction work are uncomfortably loud.
- The sounds of a fire engine siren close by are so loud that I need to cover my ears.
- The sound of screeching tires is uncomfortably loud.

These questions were removed because the wristband does not vibrate to any of these sounds, it only vibrates to speech sounds. These questions are completely out of context (and therefore unanswerable) for the scenarios in which the wristband would vibrate.

*“What was the rationale for the specifications for the audiogram?”*

*“This was simply a general inclusion criterion to make certain we were capturing garden-variety presbycusis.”*

*It would be useful for this to be mentioned.*

Response: The following sentence has now been added to the manuscript: “These specifications were chosen in order to capture individuals with hearing loss profiles in alignment with high frequency hearing loss.”

*Figure 5 appears to be truncated at the right for day 42.*

Response: Thank you for pointing this out; we have made the necessary change.

*Figure 8: Why is there a –5 label for the vertical axis?*

Response: There is an –5 on the vertical axis because the error bar for BN with HAs drops below the horizontal axis to −1.96

### Anonymous [14]:

*I appreciate the authors’ thorough revision in response to reviewer feedback, and I found this version to be very much improved. It has been a pleasure reviewing this paper and learning more about the authors’ interesting work on this novel device, which is now more clearly and thoroughly explained in this newest version of the paper.*

*I have only a few suggested minor revisions remaining as follows:*

- *In the Results section of the Abstract, it says “those without hearing aids showed a 10.78 point greater drop in average APHAB benefit score at 6 weeks.” I believe this should read 10.78 higher APHAB benefit score. It would be a drop in score from baseline to the 6-week score if discussing the global APHAB score, but if discussing the benefit score, then the score increased from baseline to 6 weeks.*

Response: Thank you for catching this; we have revised the sentence: “Those without hearing aids showed a 10.78 point larger improvement in average APHAB benefit score at 6 weeks than those with hearing aids.”

- *In the Results section of the Abstract, most of the results are discussed as the group average, with only one result framed in terms of non-HA users versus HA users. It might be helpful to more clearly specify that when the average results are presented—it is across all participants. I do not have a strong preference on this, just something I noticed.*

Response: Thank you for the suggestion, we have reworded the Results portion of the abstract for further clarity:

“By the end of the 6 week study, the average APHAB benefit score across all participants reached 12.39 points from a baseline of 40.32 to a final score of 27.93 (SD=13.11, *n*=16, *P*=.002, two-tailed dependent t-test). Those without hearing aids showed a 10.78 point larger improvement in average APHAB benefit score at 6 weeks than those with hearing aids (t(14)=2.14, *P*=.10, two-tailed independent t-test). The average benefit score across all participants for ease of communication (EOC) was 15.44 (SD=13.88 , *n*=16, *P*<.001 , two-tailed dependent t-test). The average benefit score across all participants for background noise (BN) was 10.88 (SD=17.54, *n*=16, *P*=.03, two-tailed dependent t-test). The average benefit score across all participants for reverberation (RV) was 10.84 (SD=16.95, *n*=16, *P*=.02, two-tailed dependent t-test).”

- *In the last paragraph of the Introduction, it says “...can help individuals with high frequency hearing loss to feel more confident in their ability to understand speech communication.” Although I understand why the authors are making this inference from the APHAB, it does not feel quite supported enough to jump from the APHAB results to a statement about participants’ confidence. I would strongly suggest editing this to be in line with the language used throughout the rest of the paper (eg, increasing subjective assessment of speech ability, increasing self-rated communication ability, or decreasing self-perceived hearing difficulty in daily communications).*

Response: We have revised this sentence: “In this study, we aimed to demonstrate that a simple wearable sensory substitution device that transforms speech sounds into haptic vibrations on the wrist can help individuals with high frequency hearing loss perceive a greater ability to understand speech communication throughout their normal daily routine.”

- *At the end of the APHAB section under Tasks, where it says “Higher benefit scores indicate...,” I would also suggest adding the calculation for the benefit score as unaided – aided; then, it could be deleted from the next section.*

Response: This information is already contained in the paragraph: “The test was administered through an online questionnaire that captured the data onto a datasheet for analysis. The benefit score is calculated by subtracting the final aided score at the conclusion of the trial from the baseline unaided score that was measured at the beginning of the trial. Lower raw APHAB scores indicate lower levels of disability associated with hearing loss. Higher benefit scores indicate more perceived benefits from intervention.”

- *In Table 1, I would suggest adding a column to indicate which participants had a professional hearing test and which used the app option.*

Response: Thank you for the suggestion. We have updated the table and the caption below it.

“Table 1. Demographic data. Hearing loss values are decibels of hearing loss at six pure tones in the left and the right ears. Hearing loss values are measured without cochlear implants or hearing aids. Note that 90 dB of hearing loss is the most the test can detect. Audiogram source indicates where the audiogram originated from. Audiologist indicates the audiogram was measured by an audiologist and mobile app indicates the participant provided two audiograms measured by the Mimi and Hearing & Ear Age Test Mobile apps.”

- *For the Table 2 legend, I would suggest specifying how precision and recall are calculated in terms of true positives, false positives, etc. Additionally, it would be helpful to know how the F*_*1*_ *-score is calculated.*

Response: We have updated the caption under the table to include the equations for precision, recall, and *F*_1_-score.

“Table 2. Algorithm performance. Precision is the ability of a classification model to return only the data points in a class. It is calculated by dividing the true positives by the sum of the true positives and false positives. Recall is the ability of a classification model to identify all data points in a relevant class. It is calculated by dividing the true positives by the sum of the true positives and false negatives. F1 Score is a single metric that combines recall and precision using the harmonic mean. It is calculated by dividing the true positives by the sum of the true positives plus one half of the sum of the false positives and false negatives.”

- *In the Results section comparing non-HA users to HA users, the sentence about the 10.78-point difference could be made clearer if it specified that the non-HA users had a 10.78-point higher benefit score than the HA users (rather than just saying there is a difference).*

Response: Thank you for the suggestion, we have revised this sentence: “Results showed a 10.78 point greater APHAB benefit score at 6 weeks for participants who did not use hearing aids than for participants who did (t(14)=2.14, *P*=.10, two-tailed independent t-test, Figure 7).”

- *In the same section of the Results, it says “...average APHAB benefit over baseline...”—since the benefit score reflects a reduction in the APHAB score, I would suggest framing benefit not as being “over baseline” but rather “from baseline.”*

Response: Thank you for the suggestion, we have revised this sentence: “The subgroup that did not wear hearing aids ended the study with an average APHAB benefit from baseline of 18.45 points (SD=11.70, *n*=7, *P*=.005, two-tailed dependent t-test). The subgroup that wore hearing aids ended the study with an average APHAB benefit from baseline of 7.67 points (SD=12.730, *n*=9, *P*=.11, two-tailed dependent t-test).”

- *In the Discussion section, where it says “Out of 16 participants, 14 ended the study with an APHAB score of 40 or below....” I think this would be more helpful if it said how many of them started the study with a score of 40 or below. I do not have a strong preference, however. Now that individual data are presented, it is much easier to contextualize the results.*

Response: Thank you for the suggestion; we believe this information can be extracted from the individualized data table provided in Table 1 of the final paper.

- *In the Discussion section, it says “It is also possible that participants who started the study with a lower APHAB score had more room for improvement.” I think this should say a higher APHAB score, as higher scores mean more perceived difficulty.*

Response: Thank you for the suggestion, we have revised this sentence: “It is also possible that participants who started the study with a higher APHAB score had more room for improvement, as higher APHAB scores indicate a higher degree of perceived disability.”

- *In the Conclusion, it mentions that the study was underpowered to detect the difference between HA users and non-HA users at P<.05. This is presented for the first time in the Conclusion, which seems out of place. I would suggest first mentioning this in the Limitations section above. It could also be mentioned in the Conclusion, though, because it’s an important point—but reading new information in the conclusion was a bit jarring.*

Response: Thank you for pointing this out. We have added this information to the Discussion section in the paragraph describing the possible differences for HA and non-HA users. There is also a mention of the failure to reach statistical significance in the Results section:

“Participants without hearing aids demonstrated a trend toward higher self-reported benefit from vibrotactile sensory substitution for speech understanding, though this did not reach statistical significance. Given that this group started the study trending toward a higher APHAB score (above), we presume the difference is because the hearing aid group already gains benefit from their technology and therefore has less room for improvement. It is difficult to predict what the interaction between hearing aids and vibrotactile feedback will be because of the differing signal processing techniques used in digital hearing aid technologies Digital hearing aids convert sound waves into numerical codes before amplifying them. This code contains information about a sound’s frequency and amplitude, allowing the hearing aid to be specially programmed to amplify some frequencies more than others. Digital sound processing capabilities allow an audiologist to adjust the hearing aid to a user’s needs and to different listening environments. Digital hearing aids can also be programmed to focus on sounds coming from a specific direction. It is possible the wristband represents sounds that differ significantly from those represented by the hearing aid. Future studies can possibly explore directly connecting the wristband to the user’s hearing aids through a bluetooth signal so that the wristband’s signals directly correspond with the sounds the user is hearing. For this study, the small sample size rendered the study underpowered to detect differences between those who used hearing aids and those who did not at *P*<.05. Future studies will be designed to further investigate this finding.”

#### Very Minor Comments

- *First paragraph under APHAB under Tasks, suggest revising “they are asking” to “they ask”*

Response: Thank you for this suggestion, we have revised the sentence: “These questions were removed because they ask about the unpleasantness of sounds heard through a hearing aid, which does not apply for our device.”

- *In the same section, suggest revising the two instances of “was referring” to “referred”*

Response: Thank you for the suggestion, we have revised this sentence as well: “If the participant regularly wore hearing aids, ‘with the wristband’ referred to wearing the wristband in addition to their hearing aids and ‘without the wristband’ referred to wearing their hearing aids alone.

- *For the Results section that discusses the BN score, it should read “16.99 points higher than those with hearing aids” (“with” is missing)*

Response: Thank you for pointing this out; we have made this correction: “The average BN benefit for those without hearing aids was 16.99 points higher than those with hearing aids (t(14)=2.14, *P*=.05, two-tailed independent t-test).”

## Abbreviations

APHAB: Abbreviated Profile of Hearing Aid Benefit
BN: background noise
EOC: ease of communication
HA: hearing aid
